# Early Resveratrol Treatment Mitigates Joint Degeneration and Dampens Pain in a Mouse Model of Pseudoachondroplasia (PSACH)

**DOI:** 10.3390/biom13101553

**Published:** 2023-10-20

**Authors:** Jacqueline T. Hecht, Alka C. Veerisetty, Debabrata Patra, Mohammad G. Hossain, Frankie Chiu, Claire Mobed, Francis H. Gannon, Karen L. Posey

**Affiliations:** 1 Department of Pediatrics, McGovern Medical School, The University of Texas Health Science Center at Houston (UTHealth), Houston, TX 77030, USA; jacqueline.t.hecht@uth.tmc.edu (J.T.H.); alka.veerisetty@uth.tmc.edu (A.C.V.); mohammad.g.hossain@uth.tmc.edu (M.G.H.); frankie.chiu@uth.tmc.edu (F.C.); 2 Department of Developmental Biology, Washington University School of Medicine, St. Louis, MO 63110, USA; debabratapatra@wustl.edu; 3 Department of Biology, Rice University, Houston, TX 77005, USA; com27@case.edu; 4 Departments of Pathology and Immunology and Orthopedic Surgery, Baylor College of Medicine, Houston, TX 77030, USA; fgannon@bcm.edu

**Keywords:** cartilage oligomeric matrix protein, COMP, autophagy, resveratrol, dwarfism, chondrocyte, articular cartilage, joint degeneration, joint pain

## Abstract

Pseudoachondroplasia (PSACH), a severe dwarfing condition associated with early-onset joint degeneration and lifelong joint pain, is caused by mutations in cartilage oligomeric matrix protein (COMP). The mechanisms underlying the mutant-COMP pathology have been defined using the MT-COMP mouse model of PSACH that has the common D469del mutation. Mutant-COMP protein does not fold properly, and it is retained in the rough endoplasmic reticulum (rER) of chondrocytes rather than being exported to the extracellular matrix (ECM), driving ER stress that stimulates oxidative stress and inflammation, driving a self-perpetuating cycle. CHOP (ER stress signaling protein) and TNFα inflammation drive high levels of mTORC1 signaling, shutting down autophagy and blocking ER clearance, resulting in premature loss of chondrocytes that negatively impacts linear growth and causes early joint degeneration in MT-COMP mice and PSACH. Previously, we have shown that resveratrol treatment from birth to 20 weeks prevents joint degeneration and decreases the pathological processes in articular chondrocytes. Resveratrol’s therapeutic mechanism of action in the mutant-COMP pathology was shown to act by primarily stimulating autophagy and reducing inflammation. Importantly, we demonstrated that MT-COMP mice experience pain consistent with PSACH joint pain. Here, we show, in the MT-COMP mouse, that resveratrol treatment must begin within 4 weeks to preserve joint health and reduce pain. Resveratrol treatment started at 6 or 8 weeks (to 20 weeks) was not effective in preventing joint degeneration. Collectively, our findings in MT-COMP mice show that there is a postnatal resveratrol treatment window wherein the inevitable mutant-COMP joint degeneration and pain can be prevented.

## 1. Introduction

COMP is a large pentameric, matricellular protein that binds to ECM proteins including collagen types I, II, IX, XII, XIV, matrilin-3, aggrecan, sparc, and fibronectin, providing an interaction hub [1,2,3] that contributes to cartilage homeostasis [1,2,3,4]. Mechanical loading increases COMP levels in tendons while aging or overuse decreases content, suggesting that COMP plays a role in the mechanical strength of ECM tissues [2,5]. Chondrogenesis and chondrocyte proliferation are stimulated by COMP, demonstrating that it has multiple functions [6,7]. In contrast, mutations in COMP cause pseudoachondroplasia (PSACH), a severe dwarfing condition characterized by disproportionately short stature, short limbs, joint laxity, pain, and early-onset joint degeneration [3,8,9,10,11,12,13,14,15,16,17,18]. PSACH birth parameters are within the normal range, and the first noticeable change is linear growth deceleration starting around one year of life and a waddling gait that appears after two years [19]. Characteristic rhizomelic shortening of long bones, small abnormal and underossified epiphyses, widened and irregular metaphyses, and platyspondyly are the radiograph findings that confirm the diagnosis [13,17,19]. Genetic testing is now used to confirm the clinical diagnosis. While the loss of linear growth is the most apparent feature of PSACH, joint dysfunction and pain are the most debilitating and long-term complications that diminish quality of life [20]. Significant pain begins in childhood and is most likely caused by inflammation related to the growth plate chondrocyte pathology [9,13,21,22]. Pain in adulthood is related to degenerative changes in the joints that necessitate hip replacement between the second and third decades of life [11,19]. Non-surgical treatment is critically needed since all joints are affected, especially hips, elbows, and shoulders, but not all joints are replaceable [13,20,23].

Massive retention of a lamellar material was first identified in electron microscopy (EM) images of PSACH growth plate chondrocytes from children aged 5 to 12 years, and after gene discovery, it was proven to be COMP [9,13,17]. Subsequent studies showed that an intracellular matrix was present in the enlarged ER cisternae, composed of COMP, types 2 and 9 collagens, and matrilin-3 [22]. Intracellular retention of COMP results from abnormal protein folding related to the loss of the calcium scaffold essential for proper 3-dimensional conformation [19]. To define the mechanisms that underlie the PSACH pathology, we generated the MT-COMP mouse that expresses mutant human D469del-COMP in tissues expressing type 2 collagen with doxycycline (DOX) administration that recapitulates both the clinical phenotype and PSACH chondrocyte pathology [19]. Using the MT-COMP mouse, we showed that the accumulation of mutant-COMP in the rER is toxic to the growth plate and articular chondrocytes [13,22,24]. The retention of mutant-COMP induces ER stress along with oxidative stress and inflammation, creating a self-perpetuating stress loop that leads to DNA damage and a blockage of autophagy [25]. The autophagy blockade is stimulated by increases in mTORC1 signaling driven by TNFα and CHOP [25]. mTORC1, a master regulator of growth, responds to nutritional status, cellular stress, and growth factors regulating general protein translation and autophagy [26]. Elevated mTORC1 signaling favors general protein synthesis at the expense of autophagy, which may exacerbate ER stress and directly inhibit autophagy clearance of the ER in chondrocytes [25,27]. The cumulative stress coupled with the block of ER clearance drives a senescent phenotype in articular chondrocytes that likely propagates degenerative changes to nearby cells and tissues of the joint [28]. Previously, we showed that resveratrol treatment from birth prevents mutant-COMP accumulation by stimulating autophagy and reducing inflammation and chondrocyte death, avoiding premature joint degeneration [27,29]. This work builds on the successful resveratrol joint-sparing treatment in the MT-COMP mice by defining the resveratrol treatment window that prevents MT-COMP joint degeneration.

## 2. Materials and Methods

### 2.1. Bigenic Mice

MT-COMP mice are a bigenic inducible mouse line that contains two plasmids: pTRE-COMP [22] (human COMPD469del + FLAG tag coding sequence driven by the tetracycline responsive element promoter) and pTET-On-Col II (rtTA coding sequence driven by a type II collagen promoter). These two constructs together express mutant-COMP with the D469del mutation in the cartilage of mice in the presence of DOX [22,30]. All mice were PCR genotyped to verify the presence of both transgenes [22]. DOX (500 ng/mL) was administered to mice at birth, through mother’s milk, and collected in their drinking water. Human mutant-COMP is expressed in addition to endogenous mouse COMP. This study complied with the Guide for the Care and Use of Laboratory Animals, eighth edition (ISBN-10, 0-309-15396-4), was approved by the Animal Welfare Committee at the University of Texas Medical School at Houston, and complies with NIH guidelines. Importantly, transgenic mice overexpressing wild-type COMP are similar to C57BL/6 control mice [30] and therefore are used as controls. To control for untoward outcomes related to DOX, C57BL/6 mice (control) on DOX are included in all experiments [28,30].

### 2.2. Resveratrol Administration

Resveratrol (250 mg/L [19,27,31]) was administered through drinking water starting at the following time points: birth, 4, 6, 8, 16 to 20 weeks of age (Nature’s Answer Alcohol-Free Resveratrol Reserve, Hauppauge, NY, USA). Prior to weaning, from birth to 3 weeks, resveratrol was transferred to pups through mother’s milk. Mass spectroscopy confirmed the resveratrol concentration.

### 2.3. Ibuprofen Administration

Ibuprofen gel capsule (200 mg) was diluted into 1 L of water with 500 ng/mL DOX for a final concentration of 0.2 mg/mL [32]. Water consumption was monitored to ensure that mice consumed sufficient amounts of liquid prior to using ibuprofen DOX water in experiments. Mice were provided ibuprofen DOX water 24 h prior to grooming assessment.

### 2.4. Immunohistochemistry

Hind limbs from male and female MT-COMP and C57BL\6 control mice were collected and articular cartilage analyzed, as previously described [22,30]. Briefly, hind limbs were fixed in 4% PFA, and heat-induced epitope retrieval antigen retrieval was performed with sodium citrate buffer for pS6 (Cell Signaling Technology, Danvers, MA, USA; 2215S rabbit polyclonal, 1:200), tumor necrosis factor α (TNFα) (Abcam, Cambridge, United Kingdom; ab6671, 1:200), and MMP13 (Abcam ab39012, 1:50) antibodies. With IL-6 (Bioss Woburn, MA bs-0781R, 1:200), SIRT1 (Abcam 32441, 1:100), TRAIL (Abcam ab42243, 1:200), p16 INK4a (Abcam ab189034, 1:200), MID1 (Abcam ab-70770, 1:200), CHOP (Santa Cruz SC-575; 1:100), and human COMP (Thermofisher, Waltham, MA, USA; 1-20221, 1:100) antibodies, the hindlimbs were fixed in 95% ethanol, and pepsin (1 mg/mL in 0.1 N HCl) was used for antigen retrieval. Species-specific biotinylated secondary antibodies were used for 1 h. at RT. Sagittal sections of the same thickness (5 μm) were then washed and incubated with streptavidin, horseradish peroxidase (HRP), and DAB as the chromogen. The sections were dehydrated and mounted with cytoseal 60 (Thermofisher) and then visualized under a BX51 inverted microscope (Olympus America, Center Valley, PA, USA). Limbs were fixed in 10% wt/vol formalin for terminal deoxynucleotidyl transferase-mediated deoxyuridine triphosphate-biotin nick end labeling (TUNEL) staining using the Promega G3250 kit following the manufacturer’s protocol. For proteoglycan stains, samples were deparaffinized, hydrated in distilled water, and stained with safranin-O (Spectrum Chemical, New Brunswick, NJ, USA, 477-73-6) according to the manufacturer’s protocol. All assays were performed on 10 animals in each group.

### 2.5. Joint Degeneration Scoring

A joint scoring system that quantifies early degenerative changes in the proteoglycan content of the articular cartilage of the femur and tibia, the degree of synovitis, and bone/cartilage damage was developed and employed in this study [29]. This method is more sensitive to early joint changes than the OARSI scoring system, which best describes end-stage joint damage (Figure S1). Joint degeneration scoring was performed on 5 μm sagittal sections from 10 different mice. Only sections that contained both menisci were scored in order to ensure sections were obtained from the same area of the joint. While OARSI scoring covers a wide range of OA pathology, in this study, joint degeneration scoring was modified to optimize the evaluation of early joint pathology. Four areas—synovium, bone/cartilage, tibial, and femoral articular cartilage—were scored from 0 to 3 on each Safranin-O-stained section. This early-stage joint pathology scoring system was developed specifically after examining the pathology of MT-COMP mice to quantify the early degenerative characteristics. To show the utility of this scoring system, control and MT-COMP mice were scored using OARSI scoring, OARSI scoring + meniscus/synovium component, and the early joint pathology score described here (Figure S1). All scoring systems showed a trend towards a higher level of damage for the MT-COMP joints. To show the utility of this scoring system, control and MT-COMP mice were scored using OARSI scoring, OARSI scoring + meniscus/synovium component, and the early joint pathology score described here (Figure S1). All scoring systems showed a trend towards a higher level of damage for the MT-COMP joints compared to controls, but only the early scoring system showed a significant difference. A score of 0 indicated normal or no damage, 1 = mild damage, 2 = moderate damage, and 3 = severe damage. Synovitis, bone/cartilage damage, proteoglycan of the tibia, and proteoglycan of the femur were scored individually, and all scores were summed with a maximal damage score of 12. Synovitis was defined as a mild—increase in thickness of synovial lining and increase in stromal area; a moderate—increase in stromal density; or a severe—thickening of synovial lining with further increase of stromal cellular density. Bone/cartilage damage was defined as: normal—the surface was smooth; mild—minor erosion of the surface; moderate—the presence of remodeling with minor erosion; or severe—major erosion. Proteoglycans of the articular cartilage of the tibia and femur were classified as: normal—if staining was even through to the subchondral bone; mild—when staining was thinned; moderate—thinning of the proteoglycan-stained layer and absence of staining in some areas; or severe—the widespread loss of proteoglycan staining. Ten mice per experimental group were used for each time point, providing 80–90% power to detect a minimal difference of 2 or 3 units. All scoring was performed blindly. Limb section depth, thickness, fixation, and decalcification conditions were identical. A *t*-test was used to evaluate the total joint degeneration score across six experiment groups, comparing MT-COMP to all other groups. The Kruskal-Wallis test was used to evaluate the distribution of individual scored features across six experiment groups, and the post-hoc Dunn’s test compared MT-COMP to controls.

### 2.6. Voluntary Running

**Figure 1:**
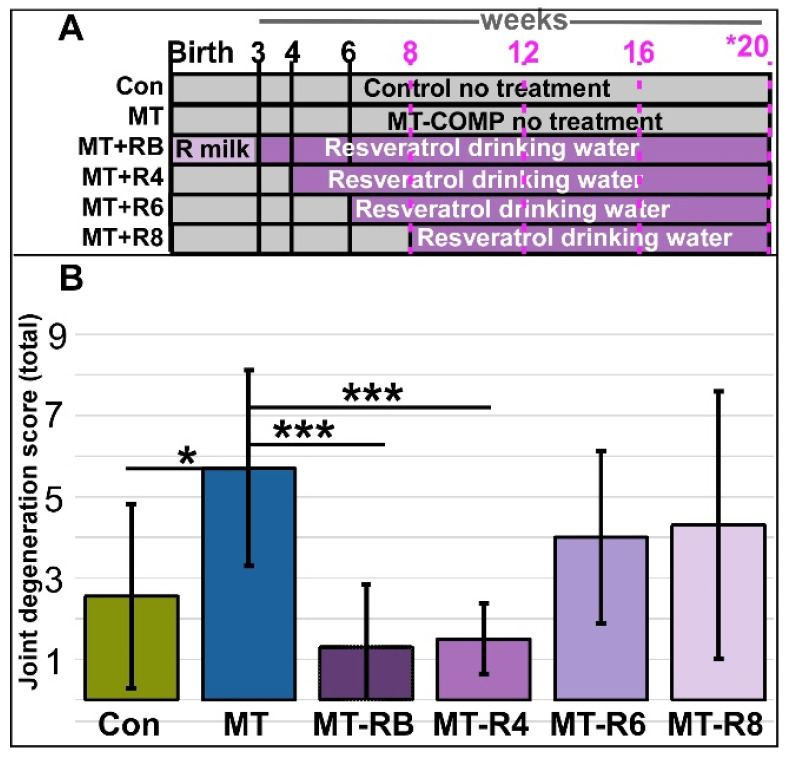
The joint degeneration score in MT-COMP mice was decreased with resveratrol treatment from birth or from 4 to 20 weeks. (**A**) Experiment design graphic. Male mice were administered DOX from birth to induce the MT-COMP phenotype and treated with resveratrol starting either at birth (MT-RB), 4 (MT-R4), 6 (MT-R6), or 8 (MT-R8) weeks until 20 weeks and analyzed. Timepoints noted in pink denote grooming evaluations. (**B**) Joint degeneration scoring total is the sum of four scores based on scoring each area from 0–3 (synovitis, femoral proteoglycans, tibial proteoglycans, and cartilage/bone damage). Results were compared to untreated MT-COMP (MT) mice at 20 weeks. MT-COMP mice treated from birth to 4 weeks had significantly improved joint degeneration scores. Ages shown in purple text denote grooming timepoints and asterisk * denotes age at which joint degeneration was evaluated. (Abbreviations: R milk = resveratrol through mother’s milk; Control = Con; MT-COMP = MT; MT-COMP mice treated with resveratrol at birth = M+RB; 4–20 weeks = M+R4; MT-COMP mice treated with resveratrol 6–20 weeks = M+R6). *n* = 10, * *p* < 0.05; *** *p* < 0.0005 (Kruskal-Wallis).

Mice were acclimated to the running wheel for 3 days before data collection. Running activity was collected on male mice for 7 days and nights. The cumulative running distance at night was calculated and compared to controls using a *t*-test. The standard deviation is represented by error bars in Figure 1.

### 2.7. Grooming Assay

Fluorescent dye (50 μL) was applied on the back of the neck equidistant between ears at time 0, and grooming efficiency was assessed 4 h later in male mice. Animals were imaged and compared to the scoring rubric previously published, with maximal grooming scoring at five [33]. The average grooming scores were analyzed with Kruskal-Wallis with a post-hoc Dwass-Steel-Critchlow-Fligner (DSCF) pairwise test. The standard deviation is represented by error bars in Figure 1.

## 3. Results

### 3.1. Resveratrol Treatment Starting at 4 Weeks Preserves Joint Health

To identify the resveratrol postnatal joint degeneration prevention window, MT-COMP mice were administered resveratrol from birth at 4, 6, 8, and 12 to 20 weeks. Joint degeneration was quantified using a 0–3 scoring system, signifying no damage to severe damage in four regions: (1) cartilage/bone degeneration, (2) proteoglycan loss in the tibia, (3) proteoglycan loss in the femur, and (4) the presence of synovitis. The joint degeneration score is the sum of the four scored regions, with a maximal score of 12. In the knee joint, early degenerative changes include synovitis, meniscus damage, and the loss of proteoglycans. This early scoring system was developed to measure degenerative changes observed in safranin-O-stained sections of MT-COMP mice knee joints at 20 weeks that are not measurable with OARSI [34] or OARSI with synovitis scoring systems (see Section 2; Supplemental Figure S1).

MT-COMP mice administered resveratrol for 12–20 weeks were indistinguishable from untreated MT-COMP mice and were not further evaluated. The total joint degeneration score of 1.5 ± sd 0.9 (±standard deviation = sd) from MT-COMP mice treated with resveratrol from 4 to 20 weeks was significantly lower than the score of 5.7 ± 2.4 from untreated MT-COMP. This was also lower than the score of 4.0 ± sd 2.1 for resveratrol-treated MT-COMP mice from 6–20 weeks or a score of 4.3 ± sd 3.3 from 8–20-week mice (Figure 1 and Supplemental Figure S2). Proteoglycan loss, an early indicator of joint damage, is a part of this score [35,36,37]. Proteoglycan loss in the articular cartilage of femur and tibia MT-COMP mice treated with resveratrol from 4 to 20 weeks was significantly less (femur 0.3 ± 0.6 and tibia 0.1 ± 0.3) compared to untreated MT-COMP mice (1.8 ± 0.9; 1.2 ± 0.6). Synovitis, which makes up a substantial portion of the total score of untreated MT-COMP mice (1.8 ± 0.6 out of a total score of 5.7 ± 2.4), was significantly reduced in MT-COMP mice treated with resveratrol from birth to 20 weeks (0.5 ± 0.5) or 4 to 20 weeks (0.4 ± 0.6). These results suggest that both synovitis and proteoglycan loss are valuable markers for the analysis of PSACH joint pathology.

### 3.2. Pain Observed in MT-COMP Mice Was Mitigated by Resveratrol Treatment

**Figure 2:**
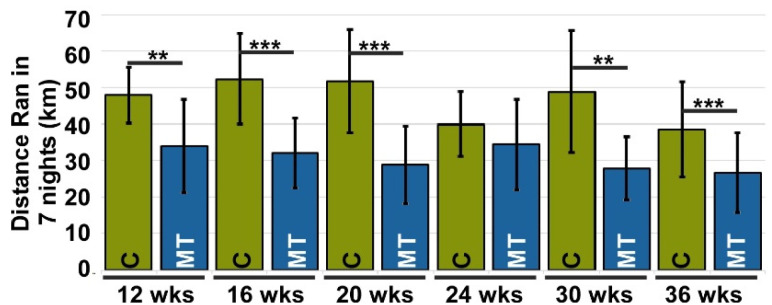
Voluntary running is reduced in MT-COMP mice, suggesting pain. All male mice were administered DOX from birth to induce mutant-COMP expression until the completion of the study. Voluntary running was used as a proxy for pain. Voluntary running data was collected at 12, 16, 20, 24, 30, and 36 weeks of age for both control C57BL\6 (C) and MT-COMP (MT) mice (*n* = 10). All mice had a 3-night acclimation period to adjust to the running wheel. MT-COMP mice ran significantly less than controls at 12, 16, 20, 30, and 36 weeks. ** *p* < 0.005; *** *p* < 0.0005 (Kruskal Wallis).

Murine pain was assessed using proxy assays evaluating alterations in instinctive behaviors [33], such as changes in physical activity (voluntary running) and grooming. Changes in voluntary running and grooming were evaluated based on the premise that pain will reduce the number of wheel rotations, or distance run, and grooming efficiency [38]. Each mouse was housed in a cage with a running wheel that measured the distance it ran. Since mice are nocturnal, voluntary running was measured over seven nights after a 3-night acclimation period. Running was assessed at 12, 16, 20, 24, 30, and 36 weeks. Figure 2 and Supplemental Figure S3 show that MT-COMP mice ran significantly less than control mice at each time point, suggesting the presence of pain.

**Figure 3:**
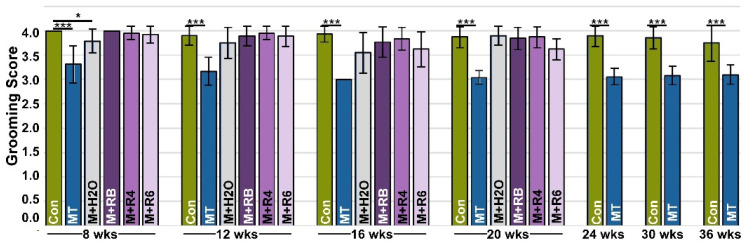
Pain is reduced with early resveratrol treatment. A grooming assay was used as a proxy for pain. The grooming assay measures the efficiency of the removal of a fluorescent dye from the fur, with a higher score indicating more effective elimination of dye (maximum score = 5). All male mice were administered DOX from birth to collection, except MT-COMP+H2O (M+H2O). Grooming was assessed at ages 8, 12, 16, 20, 24, 30, and 36 weeks in control C57BL\6 (Control) mice and MT-COMP mice, and MT-COMP mice treated with resveratrol beginning at birth (M+RB), 4 (M+4), 6 (M+6) weeks, and MT-COMP without DOX (M+H2O) (*n* ≥ 12). MT-COMP mice have a significantly lower grooming score than controls at all ages. Resveratrol treatment normalizes grooming at 8, 12, 16, and 20 weeks. Pairwise comparisons (Kruskal-Wallis) between control and all other groups are shown with asterisks. Importantly, MT-COMP grooming scores were lower than MT-COMP+H2O (MT+H2O) in the absence of the induction of mutant-COMP (*p* < 0.005 at 16 and 20 wks; *p* < 0.0005 at 8 and 12 wks). Moreover, pairwise comparisons show grooming scores from all resveratrol treatments are significantly higher than scores from untreated MT-COMP (*p* < 0.0005 at all ages tested). (Abbreviations: weeks = wks; R = resveratrol; Control = Con; MT-COMP = MT; MT-COMP mice treated with resveratrol at birth = M+RB; 4–20 weeks = M+R4; MT-COMP mice treated with resveratrol 6–20 weeks = M+R6). * *p* < 0.05; *** *p* < 0.0005.

Grooming, a natural activity, has been shown to be dampened or less efficient in the presence of pain [33]. To analyze this, we used a previously validated assay [33] where fluorescent dye was applied to the back of the neck equidistant between the ears, and the efficiency of dye removal by grooming was analyzed 4 h later. Figure 3 shows that MT-COMP mice consistently groom less than controls. To validate that grooming is associated with pain in MT-COMP mice, an analgesic (ibuprofen) was administered for 7 days, and then grooming was tested. Ibuprofen administration (0.2 mg/mL in drinking water) 7 days prior to grooming resulted in MT-COMP grooming scores comparable to those of control mice at 16 weeks (Supplemental Figure S4). Similarly, in the absence of DOX, which induces mutant-COMP expression, grooming scores were equivalent to controls (Figure 3 and Supplemental Figure S5). Importantly, MT-COMP mice administered resveratrol beginning at birth, 4, 6, and 8 weeks were groomed as efficiently as controls at all time points and better than untreated MT-COMP mice, despite some joint degeneration detected (Figure 1 and Figure 3). These results suggest that pain is not directly linked to joint degeneration levels in MT-COMP mice (Figure 1, Figure 2 and Figure 3).

### 3.3. Early Resveratrol Treatment Is Associated with a Reduction in Intracellular Comp, ER Stress, and Inflammation

**Figure 4:**
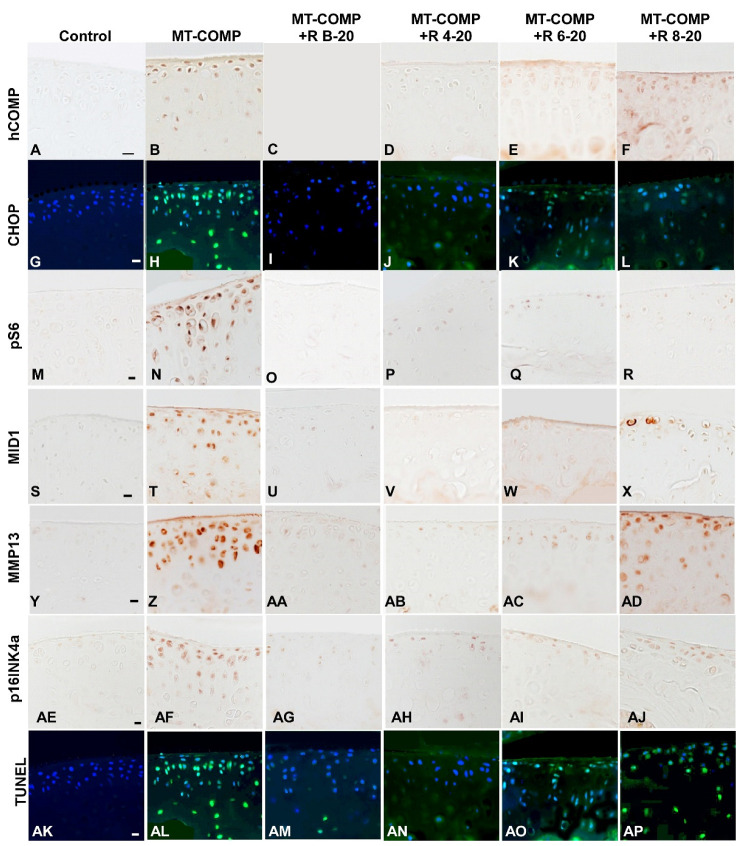
Early resveratrol treatment reduced mutant-COMP pathology in the articular cartilage of MT-COMP mice. All mice (both male and female) were administered DOX from birth to collection for analysis at 20 weeks. Resveratrol was administered from birth to 20 weeks (+R B-20), or 4 to 20 weeks (+R 4–20), 6 to 20 weeks (+R 6–20), or 8 to 20 weeks (+R 8–20). Tibial articular cartilage from control, MT-COMP, and resveratrol-treated MT-COMP mice from each group was immunostained (brown or green signal) for human COMP hCOMP (**A**–**F**), CHOP (**G**–**L**), pS6 (**M**–**R**), MID1 (**S**–**X**), MMP13 (**Y**–**AD**), pINK4a (**AE**–**AJ**), and TUNEL (**AK**–**AP**). DAPI (blue signal) shows nuclei in fluorescence images. MT-COMP articular chondrocytes show substantial intracellular hCOMP, CHOP (ER stress marker), pS6 (mTORC1 activity = blocked autophagy), MID1 (increases mTORC1 activity), MMP13 (degeneration marker), and pINK4a (senescence) signals. Resveratrol treatment for 4–20 weeks reduced MT-COMP pathology. Representative images from the analysis of 10 mice in each study group are shown. Bar (all panels) = 50 µm.

Previously, we showed that mutant-COMP retention and inflammation in articular chondrocytes start at 4 weeks, and ER stress, autophagy blockage, and articular chondrocyte death were observed at 8 and 16 weeks, respectively [28]. Our published findings demonstrated that resveratrol treatment from birth to 20 weeks eliminates mutant-COMP protein retention, ER stress, inflammation, and autophagy blockage [28]. To assess resveratrol treatment at different intervals, MT-COMP mice were administered resveratrol from birth–20, 4–20, 6–20, and 8–20 weeks and compared to no treatment and control mice. Figure 4 showed that intracellular COMP accumulation (A–F), CHOP, ER stress marker (G–L), MMP13, a cartilage degradative enzyme (Y–AD), and articular chondrocyte death (AK–AP) were not observed in MT-COMP mice treated with resveratrol from birth to 20 and 4–20 weeks but were present in those treated from 6–20 and 8–20 weeks. This data again indicates a direct correlation between pathologies and mutant-COMP accumulation. pS6, a marker of mTORC1 activity, was minimally present in all treated animals (Figure 4M–R), while MID1, a driver of mTORC1 signaling, was markedly reduced in mice treated at birth, 4, and 6 weeks (Figure 4S–X). Senescence marker p16INK4a was observed at minimal levels in a few articular chondrocytes in MT-COMP mice treated at birth, 4 weeks, and 6 weeks, and more intensely in those treated at 8 weeks (Figure 4AE–AJ). Generally, ER stress, mutant-COMP protein accumulation, and chondrocyte death occur before degeneration markers are present, suggesting that ER stress driven by mutant-COMP protein accumulation in the ER and the associated chondrocyte death is the key to stimulating MT-COMP joint degeneration processes (MMP13, p16INK4a) [25,27].

### 3.4. Resveratrol Treatment of MT-COMP Mice from 6–20 or 8–20 Weeks Correlates with the Presence of Inflammation

**Figure 5:**
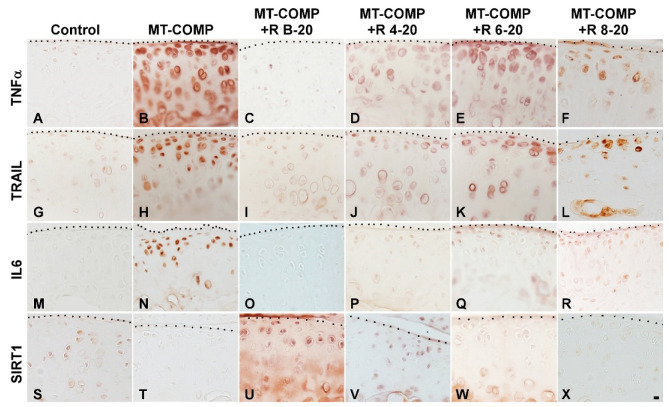
Early resveratrol treatment reduces inflammation and supports chondrocyte survival in the articular cartilage of MT-COMP mice. All mice (both male and female) were administered DOX from birth to collection for analysis at 20 weeks. Resveratrol (R) treatment was given from birth to 20 weeks (+R B-20), or 4 to 20 weeks (+R 4–20), 6 to 20 weeks (+R 6–20), or 8 to 20 weeks (+R 8–20). Tibial articular cartilage from control, MT-COMP, and MT-COMP treated with resveratrol was immunostained (brown) for TNFα (**A**–**F**), TRAIL (**G**–**L**), IL-6 (**M**–**R**), and SIRT1 (**S**–**X**). Representative images from the analysis of 10 mice in each study group are shown. Bar (all panels) = 50 µm.

We showed previously that MT-COMP mice have robust inflammatory processes and reduced levels of the pro-survival marker SIRT1 in the growth plate at 4 weeks [28,29] that were dampened by resveratrol treatment [27]. Based on those findings, inflammation was evaluated for MT-COMP mice treated with resveratrol from birth–20, 4–20, 6–20, and 8–20 weeks and compared to no treatment and control mice. Early resveratrol treatment beginning at birth or 4 weeks decreases TNFα, TRAIL, and IL6 and increases the SIRT1 signal (Figure 5A–D,G–J,M–P,S–V). Despite resveratrol treatment for 6–20 and 8–20 weeks, the MT-COMP articular cartilage had active inflammation (TNFα and TRAIL) and repressed SIRT1, a pro-survival protein, with some reduction in IL-6 (Figure 5). These findings, along with joint scoring and pain outcomes, suggest that early resveratrol treatment results in a substantial reduction in inflammatory processes, an improvement in joint/articular chondrocyte health, and a reduction in pain in MT-COMP mice.

## 4. Discussion

In this study, we show that there is a defined window of treatment, preferably immediately at birth to 4 weeks, in MT-COMP mice, after which treatment has little effect on abating the mutant-COMP pathology. Previously, we demonstrated that MT-COMP mice develop joint degeneration by 20 weeks of age, and resveratrol treatment from birth to 20 weeks prevents this pathology [28]. To define the effective postnatal resveratrol intervention window, MT-COMP mice were administered resveratrol from birth–20, 4–20, 6–20, and 8–20 weeks. Total joint degeneration scores from controls and MT-COMP mice treated with resveratrol from birth to 20 weeks were significantly lower than those from untreated MT-COMP mice (Figure 1) [27]. Resveratrol treatment that was started before retention of intracellular mutant-COMP (autophagy blockage) began and prior to the appearance of the inflammatory marker TNFα was the most effective [28]. This early resveratrol treatment at 4 weeks reduced ER stress (CHOP), mTORC1 activation (pS6/MID1), and MMP13. Resveratrol dampens articular chondrocyte stress, improves joint health as evidenced by less synovitis and bone/cartilage damage, diminishes the loss of proteoglycans in the articular cartilage, and, most importantly, reduces pain (Figure 1, Figure 2 and Figure 3). These important findings provide the first indication that there is a postnatal treatment window during which resveratrol or other treatments can mitigate joint degeneration and pain. This has important implications for the treatment of PSACH.

Our work defined a complex stress process responsible for mutant-COMP accumulation in chondrocytes that becomes cytotoxic to the cells, resulting in cell death [19,22,25,27,28,29,30,31]. COMP mutations cause protein misfolding, with 97% of the COMP pentamers expected to have at least one mutant monomer, causing massive retention of COMP in the ER [25]. The ER quality control mechanisms that should refold or degrade misfolded proteins fail, and this is evident as mutant-COMP continuously accumulates in the ER [39]. Two major pathways, autophagy and proteasomal degradation, are responsible for clearing misfolded proteins from the ER [40]. In MT-COMP mice, both ER stress and TNFα drive high levels of mTORC1 signaling [31]. Furthermore, chronic inflammation leads to reduced levels of SIRT1 in MT-COMP chondrocytes, which, in turn, exacerbates mTORC1 activity [41,42,43,44,45]. Notably, the mTORC1 signaling in MT-COMP is found to be independent of AMPK [27]. This elevated mTORC1 signaling represses autophagy, preventing mutant-COMP clearance [25]. Mutant-COMP induces ER stress through CHOP [19,30] which drives oxidative and inflammation processes that sustain a self-perpetuating cycle of stress. IL-1β and TNFα stimulate the synthesis of matrix metalloproteinases (MMPs), enzymes responsible for cleaving collagens and other proteoglycans in the extracellular matrix [46]. The elevated levels of MMP13 observed in MT-COMP articular chondrocytes are likely a consequence of chronic inflammation [28]. Oxidative stress associated with mutant-COMP generates reactive oxygen species, causing DNA damage and necroptotic chondrocyte death [19]. Chondrocyte death inhibits both long bone growth and severely compromises joint health. We previously showed that resveratrol treatment increased the number of autophagic vesicles (LC3-II positive vesicles), indicating that part of resveratrol’s therapeutic mechanism of action is the activation of autophagy, which promotes the clearance of mutant-COMP [27]. The stimulation of autophagy by resveratrol results in reduced inflammation, a decrease in cartilage-degrading enzyme MMP13 (thus mitigating degeneration), normalization of chondrocyte function by alleviating ER stress (CHOP), and relief from joint degeneration and associated pain caused by mutant-COMP. Clearance of mutant-COMP restores homeostasis and alleviates the multiple associated chondrocyte stresses.

In previous studies, we showed that resveratrol treatment from birth in MT-COMP mice dampened inflammation (TNFα, IL-1β, IL-6, and IL-18), oxidative stress, degradative enzymes (multiple MMPs), and joint damage [27,29,47,48]. IL6, TNFα, and MMP13, all of which are associated with osteoarthritis (OA) and compromised joint health, have been observed in MT-COMP joints [28,31]. IL6 is a pro-inflammatory interleukin that is intimately involved in OA joint degeneration [47,49,50]. MMP13, a degradative enzyme, plays an important role in extracellular matrix degeneration in the articular cartilage [46,51,52] and both IL-6 and TNFα stimulate MMP13 expression [53,54]. Notably, these markers of degeneration were reduced by resveratrol treatment starting at birth or 4 weeks (Figure 4 and Figure 5). In contrast, six or eight weeks of unmitigated accumulation of mutant-COMP was sufficient to drive joint degeneration in MT-COMP mice, although improved grooming scores were observed. Resveratrol treatment of MT-COMP mice from 6–20 and 8–20 weeks reduced the autophagy blockage; however, the presence of some mutant-COMP in articular chondrocytes, along with inflammation (TNFα, TRAIL), MMP13, and senescence, was sufficient to drive some joint degeneration, despite improvements in pain measurements (Figure 1, Figure 4 and Figure 5). Given that multiple pathological events in the articular cartilage downstream of mutant-COMP protein accumulation occur prior to 6 or 8 weeks of age, it is difficult to identify the precise pathological event that plays the biggest role in joint degeneration. Based on the presence of inflammatory markers and their link to degenerative enzymes, we propose that inflammation may be one driver of MT-COMP joint degeneration. Importantly, early resveratrol treatment may limit degradation through downregulation of inflammation by restraining MMP13 degradation of the extracellular matrix [46,51,52] (Figure 4).

These findings also suggest that senescent articular chondrocytes generate a senescence-associated secretory phenotype (SASP), which likely creates a pro-degenerative environment in MT-COMP joints, including the presence of MMP13 [46]. Our finding that MT-COMP mice have synovitis and damage outside the articular cartilage supports the idea that SASP may contribute to mutant-COMP joint degeneration, leading to the destruction of tissues near the articular cartilage. Resveratrol treatment from birth to 20 weeks or 4–20 weeks substantially reduced synovitis and joint pain, suggesting that reducing stress in articular chondrocytes improves overall joint health, not solely the articular cartilage. Other studies have shown that resveratrol delays joint degeneration when administered simultaneously with an OA-inducing agent [55,56], which aligns with our observation that resveratrol treatment from birth to 20 or 4–20 weeks preserves joint health. Joint degeneration after 6 or 8 weeks of mutant-COMP expression suggests that either early events predetermine joint degeneration due to the poor quality of articular cartilage or early events set in motion destructive processes that cannot be resolved after a particular threshold is exceeded, leading to articular chondrocyte death.

Several aspects of the mutant-COMP pathology, including inflammation and senescence, are particularly important to joint degeneration beyond the articular cartilage. Senescent articular chondrocytes have been observed in MT-COMP mice along with IL6 and MMP13, degenerative components of SASP [28]. These findings suggest that senescent articular chondrocytes generate SASP, which likely creates a pro-degenerative environment in MT-COMP joints [28]. Importantly, the presence of synovitis, which causes damage outside the articular cartilage, supports the idea that SASP may contribute to MT-COMP joint degeneration, leading to the destruction of tissues near the articular cartilage.

Resveratrol treatment from birth to 20 or 4–20 weeks substantially reduces synovitis and joint pain, suggesting that dampening of articular chondrocyte stress improves overall joint health. Others have shown that resveratrol delays joint degeneration when administered simultaneously with OA-induction [57,58], which aligns with our observation that resveratrol from birth to 20 or 4–20 weeks preserves joint health. Joint degeneration from 6 or 8 weeks of mutant-COMP expression suggested that early events predetermine joint degeneration, perhaps due to the poor quality of articular cartilage or early events (mutant-COMP accumulation) that set-in motion destructive processes that cannot be resolved after a particular threshold leading to articular chondrocyte death. These findings indicate that some features of mutant-COMP joint degeneration may be dampened with later resveratrol therapy, but ultimately, early intervention is essential to preserving joint health.

In PSACH, joint pain is the most debilitating complication, and similarly, MT-COMP mice show evidence of pain (Figure 2 and Figure 3). Since behavioral assays can be difficult to interpret, assessment of pain in MT-COMP mice was performed using two validated proxy assays, voluntary running [59,60,61,62] and grooming [63,64,65], both associated with instinctive behaviors. Importantly, we show resveratrol treatment reduces pain as measured by grooming. Similarly, in a clinical trial, resveratrol decreased pain in post-menopausal women [55,66], and resveratrol, when added to knee OA meloxicam therapy (anti-inflammatory), decreased pain more than meloxicam alone [56,67]. Pain is a critical aspect of modeling PSACH because it is a significant problem that often leads to joint replacements [20] and is closely intertwined with disability and decreased quality of life. Pain from joint degeneration comes either directly from damaged tissues or indirectly from inflammation or changes in tissue that alter function. Direct joint pain is associated with synovitis, meniscal damage, subchondral bone remodeling, abnormal ligament insertion, joint capsule thickening, and osteophytes [55,56]. In contrast, inflammation, cartilage degeneration, damage to the inner meniscus, and ligament laxity are indirect causes of degenerative joint pain [55,56]. Given the relatively high level of joint degeneration in the absence or decrease of pain in MT-COMP mice treated with resveratrol from 6–20 and 8–20 weeks, it is likely that resveratrol suppresses mutant-COMP-induced pain through indirect sources rather than direct ones. Any PSACH therapy must address pain. Since grooming assays indicate that resveratrol reduces pain associated with mutant-COMP pathology, this suggests that resveratrol or resveratrol in conjunction with other pain control measures could be beneficial for PSACH pain control, and early treatment may preserve joint health.

## Data Availability

Data is available upon request by writing to karen.posey@uth.tmc.edu. K.L.P. is the first and senior author.

## Supplementary Materials

The following supporting information can be downloaded at: https://www.mdpi.com/article/10.3390/biom13101553/s1, Figure S1: MT-COMP mouse joint degeneration scoring. All male mice were administered DOX from birth to collection at 20 weeks. Control (Con) and MT-COMP (MT) at 20 weeks were scored using OARSI score [28,34], OARSI score plus synovium/meniscus (syn/meni) component or the “early” OA scoring system described in this manuscript and previously used to evaluate MT-COMP mice [28,29]. Only the early joint degeneration scoring system quantified the early mild joint degeneration in MT-COMP mice, while the OARSI scoring system provided better quantification of late-stage severe degeneration. * *p* < 0.05. Figure S2: Joint degeneration score in MT-COMP mice was decreased with resveratrol treatment from birth or from 4 to 20 weeks. Male mice were administered with DOX from birth to induce the MT-COMP phenotype and treated with resveratrol starting either at: birth (MT-RB), or 4 (MT-R4), 6 (MT-R6) or 8 (MT-R8) weeks until 20 weeks and analyzed. Joint degeneration scoring total is the sum of four scores based on scoring each area from 0–3 (synovitis, femoral proteoglycans, tibial proteoglycans and cartilage/bone damage). Results were compared to untreated MT-COMP (MT) mice at 20 weeks. MT-COMP mice treated from birth and 4 weeks had significantly improved joint degeneration scores. (Abbreviations: Control = Con; MT-COMP = MT; MT-COMP mice treated with resveratrol at birth = M+RB; 4–20 weeks = M+R4; MT-COMP mice treated with resveratrol 6–20 weeks = M+R6). *N* = 10, * *p* < 0.05; *** *p* < 0.0005 (Kruskal Wallis). Figure S3: Voluntary running is reduced in MT-COMP mice suggesting pain. All male mice were administered DOX from birth to induce mutant-COMP expression until completion of study. Voluntary running was used as a proxy for pain. Voluntary running data was collected at 12, 16, 20, 24, 30 and 36 weeks of age for both controls C57BL\6 (C) and MT-COMP (MT) mice (*n* = 10). All mice had a 3-night acclimation period to adjust to running wheel. MT-COMP mice ran significantly less than controls at 12, 16, 20, 30 and 36 weeks. ** *p* < 0.005; *** *p* < 0.0005 (Kruskal Wallis). Figure S4: Ibuprofen improves grooming score of MT-COMP mice. All male mice were administered DOX from birth to analysis. Grooming was scored from control (green bar) and MT-COMP (blue bar) at 16 weeks. Another group of mice (*n* = 10) grooming was measured at 15 weeks (light blue bar MT-COMP 15 wks) ibuprofen 0.2 mg/mL [32] was administered for one week and grooming was remeasured (orange bar MT-COMP 16 wks ibup). Kruskal Wallis test was used for comparison *** *p* < 0.0005. Figure S5: Pain is reduced with early resveratrol treatment. A grooming assay was used as a proxy for pain. The grooming assay measures the efficiency of removal of a fluorescent dye from the fur, with higher score indicating more effective elimination of dye (maximal score = 5). All male mice were administered DOX from birth to collection except MT-COMP-H2O (M-H2O). Grooming was assessed at ages: 8, 12, 16, 20, 24, 30 and 36 weeks in control C57BL\6 (Control) mice and MT-COMP mice, and MT-COMP mice treated with resveratrol beginning at birth (M+RB), 4 (M+4), 6 (M+6) weeks and MT-COMP without DOX (M+H2O) (*n* ≥ 12). MT-COMP mice have a significantly lower grooming score than controls at all ages. Resveratrol treatment normalizes grooming at 8, 12, 16 and 20 weeks. Pairwise comparisons (Kruskal Wallis) between control and all other groups are shown with asterisks. Importantly, MT-COMP grooming scores were lower than MT-COMP+H2O (MT+H2O) in the absence of the induction of mutant-COMP (*p* < 0.005 at 16 and 20 wks; *p* < 0.0005 at 8 and 12 wks). Moreover, pairwise comparisons show grooming scores from all resveratrol treatments are significantly higher than scores from untreated MT-COMP (*p* < 0.0005 at all ages tested). The number of mice in each group that scored 4.0 are shown in red, 3.5 are shown in brown, and 3.0 are shown in pink. (Abbreviations: weeks = wks; R = resveratrol, Control = Con; MT-COMP = MT; MT-COMP mice treated with resveratrol at birth = M+RB; 4–20 weeks = M+R4; MT-COMP mice treated with resveratrol 6–20 weeks = M+R6. * *p* < 0.05; *** *p* < 0.0005
