# Supplementary material for: Early Resveratrol Treatment Mitigates Joint Degeneration and Dampens Pain in a Mouse Model of Pseudoachondroplasia (PSACH)
Source: Biomolecules. 2023 Oct 20;13(10):1553. doi: 10.3390/biom13101553 (PMC10605626; doi:10.3390/biom13101553)
Supplement: Supplementary file 1 [file biomolecules-13-01553-s001.zip › biomolecules-2652232-supplementary.pdf]

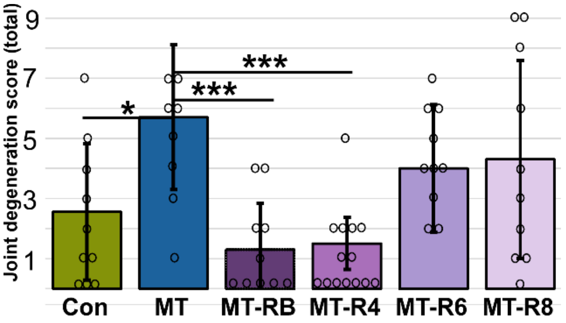

Figure S1: MT-COMP mouse joint degeneration scoring. All male mice were administered DOX from birth to collection at 20 weeks. Control (Con) and MT-COMP (MT) at 20 weeks were scored using OARSI score [28,34], OARSI score plus synovium/meniscus (syn/meni) component or the "early" OA scoring system described in this manuscript and previously used to evaluate MT-COMP mice [28,29]. Only the early joint degeneration scoring system quantified the early mild joint degeneration in MT-COMP mice, while the OARSI scoring system provided better quantifica-tion of late-stage severe degeneration. *$p < 0.05$.

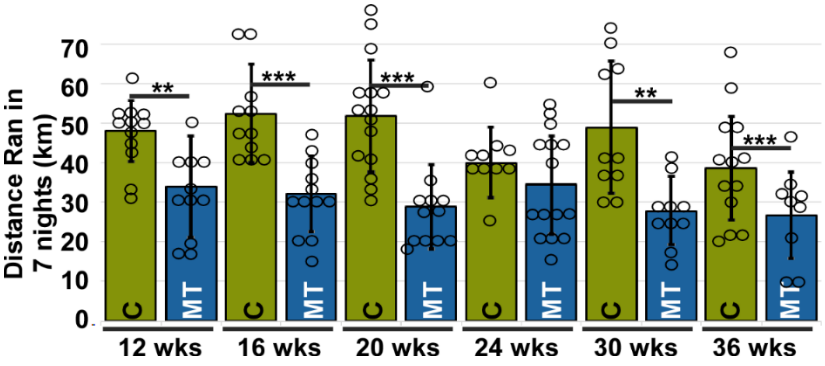

Figure S2: Joint degeneration score in MT-COMP mice was decreased with resveratrol treatment from birth or from 4 to 20 weeks. Male mice were administered with DOX from birth to induce the MT-COMP phenotype and treated with resveratrol starting either at: birth (MT-RB), or 4 (MT-R4), 6 (MT-R6) or 8 (MT-R8) weeks until 20 weeks and analyzed. Joint degeneration scoring total is the sum of four scores based on scoring each area from 0-3 (synovitis, femoral proteogly-cans, tibial proteoglycans and cartilage/bone damage). Results were compared to untreated MT-COMP (MT) mice at 20 weeks. MT-COMP mice treated from birth and 4 weeks had signifi-cantly improved joint degeneration scores. (Abbreviations: Control = Con; MT-COMP = MT; MT-COMP mice treated with resveratrol at birth = M +RB; 4-20 weeks = M+R4; MT-COMP mice treated with resveratrol 6-20 weeks = M+R6). n=10, *$p < 0.05$; ***$p < 0.0005$ (Kruskal Wallis).

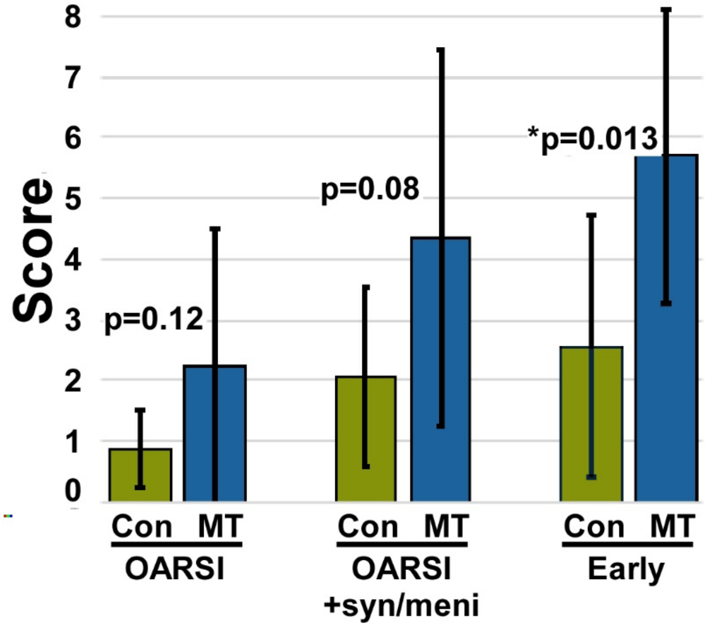

Figure S3: Voluntary running is reduced in MT-COMP mice suggesting pain. All male mice were administered DOX from birth to induce mutant-COMP expression until completion of study. Voluntary running was used as a proxy for pain. Voluntary running data was collected at 12, 16, 20, 24, 30 and 36 weeks of age for both controls C57BL\6 (C) and MT-COMP (MT) mice (n=10). All mice had a 3-night acclimation period to adjust to running wheel. MT-COMP mice ran significantly less than controls at 12, 16, 20, 30 and 36 weeks. **p < 0.005; ***p < 0.0005 (Kruskal Wallis).

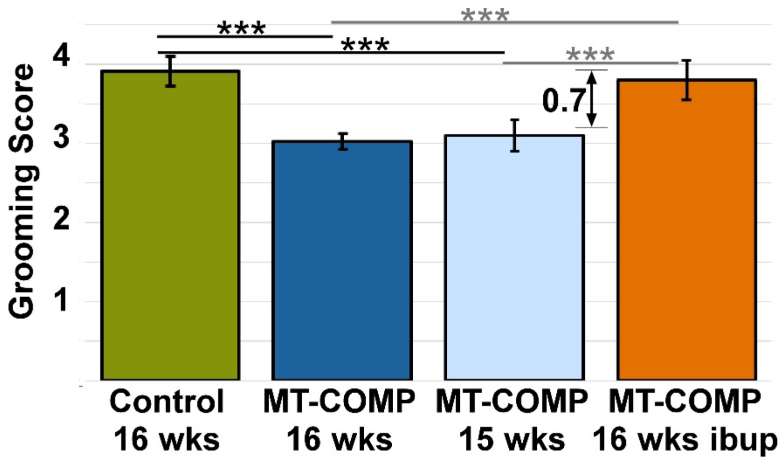

Figure S4: Ibuprofen improves grooming score of MT-COMP mice. All male mice were adminis-tered DOX from birth to analysis. Grooming was scored from control (green bar) and MT-COMP (blue bar) at 16 weeks. Another group of mice (n=10) grooming was measured at 15 weeks (light blue bar MT-COMP 15 wks) ibuprofen 0.2 mg/ml [32] was administered for one week and grooming was remeasured (orange bar MT-COMP 16 wks ibup). Kruskal Wallis test was used for comparison ***p < 0.0005.

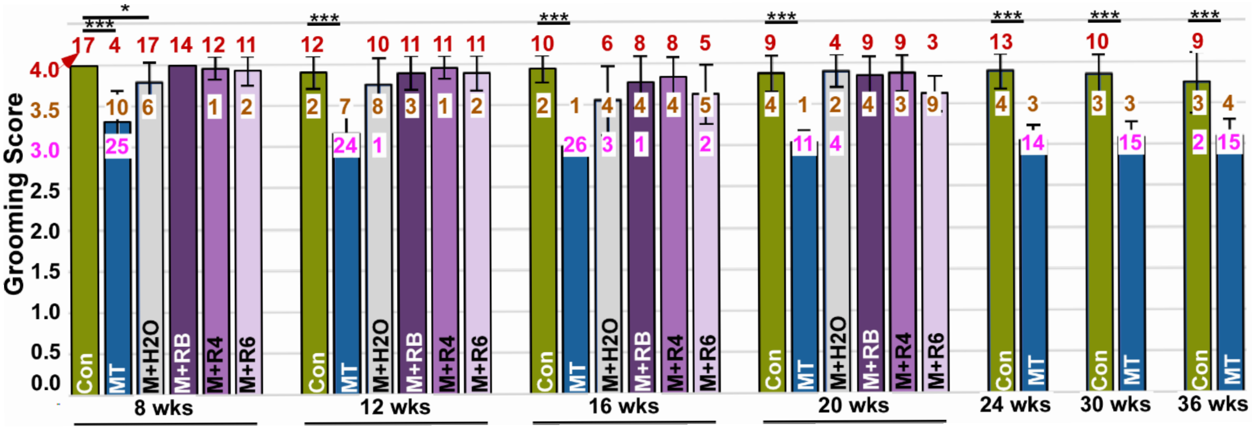

Figure S5: Pain is reduced with early resveratrol treatment. A grooming assay was used as a proxy for pain. The grooming assay measures the efficiency of removal of a fluorescent dye from the fur, with higher score indicating more effective elimination of dye (maximal score = 5). All male mice were administered DOX from birth to collection except MT-COMP-H2O (M-H2O). Grooming was assessed at ages: 8, 12, 16, 20, 24, 30 and 36 weeks in control C57BL\6 (Control) mice and MT-COMP mice, and MT-COMP mice treated with resveratrol beginning at birth (M-RB), 4 (M-4), 6 (M-6) weeks and MT-COMP without DOX (M-H2O) ($n \geqslant 12$). MT-COMP mice have a significantly lower grooming score than controls at all ages. Resveratrol treatment normalizes grooming at 8, 12, 16 and 20 weeks. Pairwise comparisons (Kruskal Wallis) between control and all other groups are shown with asterisks. Importantly, MT-COMP grooming scores were lower than MT-COMP-H2O (MT-H2O) in the absence of the induction of mutant-COMP ($p < 0.005$ at 16 and 20 wks; $p < 0.0005$ at 8 and 12 wks). Moreover, pairwise comparisons show grooming scores from all resveratrol treatments are significantly higher than scores from untreated MT-COMP ($p < 0.0005$ at all ages tested). The number of mice in each group that scored 4.0 are shown in red, 3.5 are shown in brown, and 3.0 are shown in pink. (Abbreviations: weeks = wks; R = resveratrol, Control = Con; MT-COMP = MT; MT-COMP mice treated with resveratrol at birth = M+RB; 4-20 weeks = M+R4; MT-COMP mice treated with resveratrol 6-20 weeks = M+R6. *$p < 0.05$; ***$p < 0.0005$.
